# The Dying Forward Hypothesis of ALS: Tracing Its History

**DOI:** 10.3390/brainsci11030300

**Published:** 2021-02-27

**Authors:** Andrew Eisen

**Affiliations:** Division of Neurology, Department of Medicine, University of British Columbia, Vancouver, BC V5Z 1M9, Canada; eisen@mail.ubc.ca

**Keywords:** amyotrophic lateral sclerosis, dying-forward, neurodegeneration, TDP-43, frontotemporal dementia, neural networks

## Abstract

The site of origin of amyotrophic lateral sclerosis (ALS), although unsettled, is increasingly recognized as being cortico-fugal, which is a dying-forward process primarily starting in the corticomotoneuronal system. A variety of iterations of this concept date back to over 150 years. Recently, the hallmark TAR DNA-binding protein 43 (TDP-43) pathology, seen in >95% of patients with ALS, has been shown to be largely restricted to corticofugal projecting neurons (“dying forward”). Possibly, soluble but toxic cytoplasmic TDP-43 could enter the axoplasm of Betz cells, subsequently causing dysregulation of nuclear protein in the lower brainstem and spinal cord anterior horn cells. As the disease progresses, cortical involvement in ALS becomes widespread, including or starting with frontotemporal dementia, implying a broader view of ALS as a brain disease. The onset at the motor and premotor cortices should be considered a nidus at the edge of multiple cortical networks which eventually become disrupted, causing failure of a widespread cortical connectome.

## 1. Introduction

Neurodegenerative disorders, including amyotrophic lateral sclerosis (ALS), are complex polygenic diseases, resulting in multisystem impairment of neocortical networks, primarily involving the neocortex. Specific to ALS is dysfunction of the expanded human corticomotoneuronal system [1]. This system is the anatomical infrastructure of many early clinical features of ALS, a singularly human disorder [2,3]. Early deficits include loss of vocalization requiring the integration of a complex respiratory system, impaired fractionation of digits and thumb opposability, responsible for manipulative agility, and difficulty with upright walking, especially the ability to navigate uneven and tricky surfaces. These initial symptoms reflect dysfunction of the corticomotoneuronal system [4]. In addition is the association of frontotemporal dementia (FTD), causing language impairment, failing executive function, and deteriorating socialization [5,6]. This paragraph underscores my present view that ALS is a “brain disease” [3].

**Figure 1:**
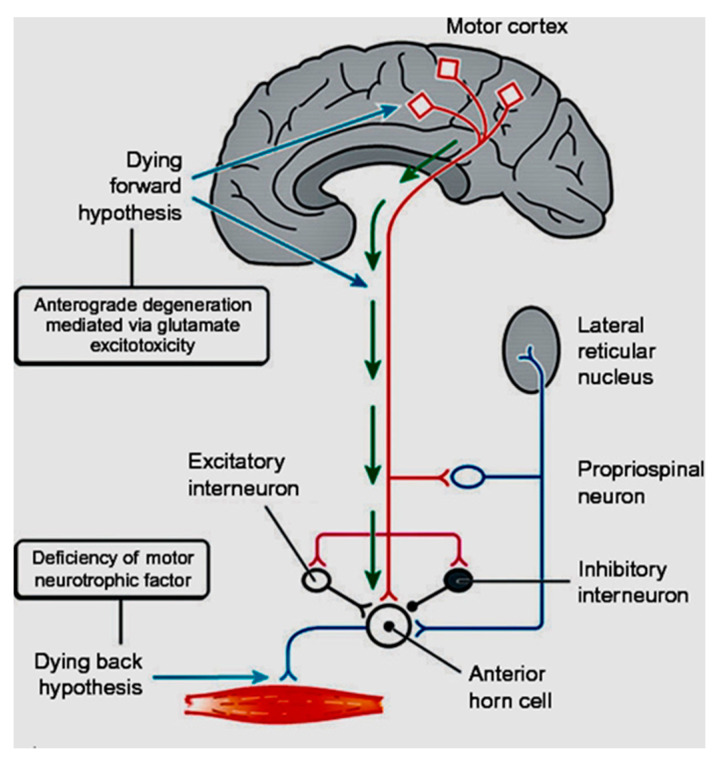
From Kiernan et al. [2]. The dying-forward hypothesis postulates that ALS commences in the motor and pre-motor cortices’ pyramidal neurons and, through antegrade mechanisms, causes dysfunction and death of the bulbar and spinal motor neurons. Excitotoxicity is important but not the only factor. The hallmark TAR DNA-binding protein 43 (TDP-43) pathology, seen in >95% of patients with ALS, is largely restricted to corticofugal projecting neurons (“dying forward”). In broader terms, this site of origin may be considered as the nidus of a spreading network disorder associated with frontotemporal dementia in ALS. In any event, ALS is best regarded as a degenerative brain disease. The figure indicates that there are alternative hypotheses of origin site which include dying-back and independent degeneration of the upper and lower motor neurons.

In coining the term “corticomotoneuronal hypothesis” [7], I first proposed some thirty years ago that ALS had its origins in the brain. This resulted in considerable controversy and many ALS physicians and scientists regarded the idea with skepticism. Though the earliest anatomical site of neurodegeneration in ALS is still not known for certain, as we enter the third decade of the 21st century, a top-down, dying-forward process, as opposed to a bottom-up, dying-back process, has become increasingly adopted. The dying-forward hypothesis proposes that glutamate excitotoxicity at the level of the cortical motor neuron ultimately results in anterior horn cell metabolic deficit. In contrast, the dying-back hypothesis proposes that ALS pathology is initiated at the level of the lower motor neurons and advances in a retrograde direction from the neuromuscular junction into the central nervous system. See Figure 1 taken from Kiernan et al. 2011 [2].

The intention here is not to review in depth evidence supporting the dying-forward hypothesis. This has been done effectively in several recent publications [8,9,10,11,12]. However, a few key points are worth emphasizing. Involvement of the motor cortex is a consistent finding in pathological studies of ALS, affecting Betz cells as well as local cortical neurons. The hallmark TAR DNA-binding protein 43 (TDP-43) pathology, seen in >95% of patients with ALS, is largely restricted to corticofugal projecting neurons (“dying forward”) [8]. Furthermore, the histological patterns of TDP-43 pathology in the motor cortex are shared in ALS and FTD, whether they occur together or independently. Both Betz cells, other pyramidal corticofugal neurons in the motor neocortex and alpha-motoneurons of the lower brainstem and spinal cord become involved at the beginning of the pathological cascade underlying ALS. There is initial evidence that whereas alpha-motoneurons lose normal nuclear TDP-43 expression followed by the formation of phosphorylated TDP-43 aggregates within their cytoplasm, Betz cells (and other pyramidal corticofugal neurons), by contrast, the loss of nuclear TDP-43 expression is largely unassociated with the development of cytoplasmic aggregations [13]. In the absence of cytoplasmic inclusions, namely, soluble and, probably toxic cytoplasmic TDP-43 could then enter the axoplasm of Betz cells and other pyramidal neurons, with transmission by axonal transport to the corresponding alpha-motoneurons in the lower brainstem and spinal cord, contributing to dysregulation of their normal nuclear protein [13]. Sophisticated MRI imaging and threshold tracking using transcranial magnetic stimulation, convincingly point to a cortical origin of ALS [14,15,16,17].

Pathological involvement of the corpus callosum in ALS has long been appreciated [18] and is a potential conduit for interhemispheric spread of pathology in ALS [19]. In humans, this structure is massive compared to other species, supporting the view that ALS is a uniquely human disease arising from selective vulnerabilities introduced by the complexity of the neocortex [20]. Sophisticated MRI imaging and threshold tracking using transcranial magnetic stimulation convincingly point to a cortical origin of ALS [17,18,19,20]. It is now clear that the brain is central to ALS. In my view, the “dying back hypothesis” is no longer tenable. This brief review traces how ALS came to be accepted as a primary disease of the human brain and in particular, its motor and pre-motor cortices.

## 2. Nineteenth Century

Jean-Martin Charcot was unambiguous that ALS started in the brain and, *“by necessity degeneration of the anterior horn cells in the spinal cord followed*” [21]. How he was able reached this conclusion, based on the pathology of only 10 patients, is somewhat of a mystery. However, he was enthralled with Betz cells, although he knew nothing of the corticomotoneuronal system. In 1875, as the subject of his annual course on Pathological Anatomy, Charcot launched a series of lectures on cerebral localization and contemporaneously used Betz as the example to illuminate the great transition in philosophy that had recently occurred regarding the brain. *“Lastly, there are the so-called pyramidal giant cells, which were carefully described by Betz and Mierzejewsky. They sometimes attain a diameter of ’040 mm to ’050 mm, that is to say, a diameter equal to that of the large ganglionic or motor cells in the anterior grey cornua of the spinal cord”* [22]. The Ukrainian anatomist and histologist, Vladimir Alekseyevich Betz (1834–94), played a pivotal role in reshaping scientific and philosophical approaches to the brain, connecting cerebral localization, function, and brain microstructure [23]. Betz revolutionized methods of cell fixation and staining, and discovered and named the giant cell within the motor cortex in 1874 [24]. Bevan Lewis [25] noted that these cells (referring to Betz cells) have a “motor significance” and “that in their configuration, size, and distribution, they present us with a thoroughly unique formation”. Nevertheless, Bevan, and subsequently Walshe [25,26,27], concluded that Betz cells were not anatomically unique, and simply reflected the largest pyramidal neurons within the primary motor cortex with corticofugal projections. The discovery of and insight into the pyramidal cells, i.e., “the cells of Betz”, allowed scattered scientific facts and findings to be systematized and became the basis for neuro-histological works of Cajal, Golgi, Lewis, Campbell, and Brodmann as well as neurophysiological and surgical studies of Broca, Horsley, Sherrington, Cushing, and Penfield [23]. Several of Charcot’s contemporaries disagreed with his view that ALS had its origin in the brain, especially Gowers who considered that the cells in the motor cortex and spinal cord died independently of each other [28].

Charcot was adamant that dementia was not a component of ALS. In this he was incorrect, and had he appreciated the overlap of ALS and frontotemporal dementia, his dogma that ALS was a brain disease might not have been buried for the next 150 years! Not too long after Charcot’s first description of ALS, Mott (1895) struggled with the issue of the site of origin of ALS [29]. *“Are we to believe that the degeneration of the upper segment of the motor path begins in the terminations of the crossed pyramidal tracts in the spinal cord and spreads gradually upwards? Or is it due to degenerative changes in the cortex leading to degeneration and atrophy of the large cells of the third layer which give origin by their axis cylinder processes to the fibers of the pyramidal tract?”* In other words, is the degeneration dying-back or dying-forward? He was unable to reach a conclusive answer. The question of site of origin of ALS was not taken up again for another 45 years. Chapter 64 of Kinnier Wilson’s two-volume textbook “Neurology”, published posthumously in 1940, is devoted to ALS. It demonstrates his extraordinary understanding of the clinical heterogeneity of the disease [30]. *“Disparity as regards Betz cell and pyramidal fibre (tract) lesions of upper motor neurones (giant cells of Betz) are usually pronounced, but, a disconcerting feature is the disparity between the respective amounts of pyramidal cell and fibre disease… Whether the process attacks cell first, or fibres, or both at once, is so undecided as to put an exclusively central origin out of the question”.* There, the site of origin of ALS died, at least in written accounts until the 1980s.

## 3. Twentieth Century

In 1988, Hudson and Kiernan [31], colleagues of mine, who were also working in Canada, wrote a rarely appreciated letter to The Lancet, “Preservation of certain voluntary muscles in motorneurone disease” [31]. They proposed *“that the extraocular muscle nuclei and pelvic sphincter neurons are spared in ALS because they are the only lower motor neurons that do not receive direct afferents from the cerebral cortex. Therefore, they would not be trans-synaptically affected by degeneration of the corticospinal tracts”.* They hypothesized the cortical neurons may normally provide trophic support for neurons with which they make contact in the anterior horn and motor nuclei of the cranial nerves and, therefore, the primary pathology of motoneurons disease might be sought in the cortex rather than the spinal cord. In an attempt to prove this idea, they were the first to compare the size/number of cortical and lower motor neurons in ALS autopsy studies [32]. *“The absence of any correlation between the severity of pathological change in the cortex and spinal cord (or brainstem) suggests that neuronal degeneration in these sites proceeds independently. The present results also fail to provide support for the idea that lower motor neuron degeneration is due to excessive responsiveness to a possibly toxic transmitter, such as glutamate.”* As a result of this negative study, Hudson abandoned any further efforts to investigate the site of origin of ALS.

There are inherent problems with autopsy correlation of corticomotoneuronal counts/size with alpha-motoneurons. The corticospinal tract (CST) has multiple functions sharing one characteristic, namely, cortical control of bulbar and spinal cord activity, through monosynaptic corticomotoneurons and other pyramidal, indirectly synapsing neurons. Most CST axons originate in cortical layer V of the primary motor and sensory cortex (M1 and S1). A smaller proportion arise from the premotor cortex, supplementary motor cortex and secondary somatosensory cortices. Corticomotoneuronal connections from fast-conducting CST fibres arise exclusively from the caudal primary motor cortex (new M1) and from area 3a [33]. But the more rostral primary motor cortex (old M1), also has corticomotoneuronal connections through more slowly conducting CST axons and through di-synaptic connections with motoneurons via interposed interneurons [34]. More recently, large pyramidal neurons (Betz cells) buried in the depth of a restricted segment of the dorsal cingulate sulcus have been described [35], a location that overlaps the ‘‘cingulate hidden hand’’ area identified by electrostimulation studies [36]. From an evolutionary perspective these Betz cells, have been considered the forerunner of the highly refined primary motor field [35,37,38]. At the spinal level, the CST terminates in the ventral horns but also projects heavily to the intermediate zone [39]. There are CST projections to the dorsal horn, traditionally viewed as the ‘sensory’ in function; these projections arise from the sensory cortex (Si) but not from M1 [40].

Over 85% of pyramidal tract fibers have a diameter < 4 mu, the diameter of the remaining fibers varying from 4–10 mu (10%) to 10–20 mu (<1.5%) [41]. The thinner fibers probably originate from areas 6 and 4 (medial and lateral), whereas the thick ones originate from area 4 only. The thickest pyramidal tract fibers are probably axons of Betz cells, which establish monosynaptic connections (corticomotoneurons) with the spinal motoneurons as far down as the lumbosacral segments of the spinal cord [42].

In humans, the fast-conducting corticomotoneurons comprise a small percentage of pyramidal tract neurons (PTNs), but the exact number is not known. They synapse with all motor neuron pools, except those to the external ocular muscles and sphincter muscles (both relatively spared in ALS) [43]. These long-range axonal corticomotoneuronal synapses distinguish primates from other mammalian species, and this system is particularly well-developed in humans. Different muscle functions are generated by separate populations of corticomotoneurons, which are widely separated within the neocortex. Corticomotoneuronal synapses in humans are widely distributed onto many functionally related anterior horn cells. For example, they may make up a ‘decisive’ proportion of the synaptic input in relation to thumb opposition and finger/hand function, with different corticomotoneuronal populations subserving precision versus power grip. Such synaptic arrangements also determine gait functions requiring finesse to explore uneven terrains and possibly in the complex functions of vocalisation, breathing and other multineuronal bulbar properties [44]. These functions seem to be uniquely vulnerable in ALS, as the split hand syndrome [45,46], the split leg syndrome [47] and more recently described split elbow syndrome [48,49]. Importantly, the numeric relationship between corticomotoneurons, their axons and anterior horn cells is not one to one. Each anterior horn cell receives input from many corticomotoneurons (convergence), and a single corticomotoneuron innervates many different anterior horn cells, usually belonging to more than one motor neuron pool, and sometimes subserving both agonist and antagonist muscle functions (divergence) [50,51]. This functional arrangement precludes meaningful autopsy correlation of corticomotoneurons and fibre tracts, and alpha-motorneurons.

## 4. The Growth of Electrophysiological Support

Prior to the latter half of the 1980s, investigative work in living ALS patients throughout much of the world was biased towards the lower motor neuron. Function and dysfunction of the ALS lower motor neuron was readily accessible, primarily using electromyography [52,53]. This lower motor neuron-centric view of ALS further emphasized the disorder being erroneously classified as a neuromuscular disease. Ability to examine the upper motor neuron in vivo before the 1990s was limited, and clinical assessment of upper motor neuron deficits in ALS continues to be fraught with difficulties [54,55]. Now that it is unanimously accepted that ALS is a multisystem disorder, with prominent cortical involvement, a view fully supported by overwhelming evidence from psychometric, imaging, genetic, and pathological studies, it is untenable in the twenty-first century to continue to classify ALS as a neuromuscular disorder [56]. It should correctly be classified as a neurodegenerative disease, as was suggested two decades ago by Eisen and Calne [57].

My early formulations proposing the primacy of the brain in ALS developed from two thoughts [7,57]. First, as espoused by Hudson and Kiernan [31], related to the selective sparing of certain motor neuron pools in ALS. In particular, those anterior horn cells that do not receive direct (monosynaptic) input from the upper motor neuron (corticomotoneurons), a term initially coined by Bernhard et al. (1954). The sparing of these motor neuron pools is relative, as eventually they too are affected. However, various types of oculomotor dysfunction such as square-wave jerks, saccadic dysmetria, abnormal cogwheeling smooth pursuit, and head shaking, and positional nystagmus have been described in ALS patients at a relatively early disease stage [58]. But, since it is generally established that oculomotor motoneurons do not receive direct projections from the cortex, if ALS results from pathological processes involving such direct projections, then it is important to determine whether there is oculomotor weakness in patients with ALS. Clinical and neurophysiological findings demonstrate that while there are deficits, as those described above, these reflect problems with higher-level control and are not indicative of the motoneuron loss or weakness that characterises ALS in, for example, the hand and the foot [8].

Similarly, electrophysiological studies have shown that the external anal sphincter is not normal in ALS, but there is relative resistance sufficient to prevent incontinence, even in the longer-surviving older patients [59]. My other thought regarding the primacy of the brain in ALS arose from the fact that neurodegenerative disorders, certainly ALS, are uniquely human. There are no natural animal models that come close to human ALS. I accept the considerable value that induced animal models have and continue to play in furthering our understanding of ALS, but they cannot mimic the human disease. This, I contended, is largely related to the expanded human neocortex. In particular, the direct corticospinal projection (corticomotoneuronal system) is the latest in the development of the nervous system being associated with the least well conserved genes and therefore, most vulnerable to environmental change.

In ALS, it only became feasible to study the upper motor neuron in awake humans, in vivo, late in the 1980s. During this period, MRI studies of ALS were sparse, limited to very few patients, and the results were non-specific [60,61]. After 2000, enhanced software programs began to give insight into several aspects of ALS [62]. In the late 1980s, transcranial magnetic stimulation (TMS) started to be used to investigate the upper motor neuron in ALS, first in the United Kingdom [63] and subsequently in Canada [64,65,66]. Upper motor neuron ALS physiological studies using TMS subsequently employed increasingly sophisticated techniques: triple stimulation, peristimulus time histograms, and finally, threshold tracking developed in Australia [14,15,16,67]. This last technique has consistently shown that hyperexcitability precedes the onset of clinically overt ALS [68].

## 5. Beyond the Motor System

Although, the motor system is pre-eminent in ALS, extra-motor areas are also involved [69]. Magnetic resonance imaging (MRI) analysis of regional volumetric changes, particularly voxel-based morphometry (VBM), has demonstrated that brain atrophy occurs in frontal, temporal, and parietal lobes of both hemispheres [70,71,72]. Thus, ALS should be considered a degenerative brain disorder, extending beyond the classical motor system. Frontotemporal Dementia (FTD) is now a so well-established component of ALS [73,74], underscoring how widespread cortical involvement in ALS becomes. Clinically, up to 50% of ALS patients have some degree of cognitive or behavioral impairment, and up to 33% of patients with FTD have evidence of motor neuron involvement [75,76,77]. The discovery of hexanucleotide repeat expansion in the first intron of the C9ORF72 gene on chromosome 9p21 associated with both ALS and FTD further supports their shared origins [78,79]. In addition, evolutionary and early life developmental concepts, as they relate to the clinical deficits in ALS, also support early diffuse cortical involvement in ALS [20].

Brain networks are characterized in graph theory as comprising nodes (brain regions) and edges (connections). The structural integrity of the motor network and the way it is embedded in the overall brain network underlies proper motor functioning. Even though brain network analyses have been extensively applied to Alzheimer’s disease, in ALS, the field is still in its infancy [80] and structural covariance networks in ALS have only recently been explored. Given that ALS is probably driven by cortical pathologies [8], structural covariance network analysis based on gray matter morphology may provide new insights into the pathophysiology of this disease. A positive correlation between functional connectedness of the motor network and disease progression rate has been described, suggesting spread of disease along functional connections of the motor network [81]. Furthermore, white matter degeneration in ALS is linked to the motor cortex, and impaired structural networks have a strong correspondence to affected white matter tracts identified using more conventional voxel-based methods [82].

## 6. Conclusions

There are those that advocate that in ALS, there is independent demise of the upper and lower motor neurons [83]. However, these two neurons are intricately related and continuously “talk to each other”. The “dying forward hypothesis” I proposed 30 years ago is attractive on many fronts and has over time gained considerable support. The restrictive format originally conceived needs expansion as clearly much of the neocortex is involved in ALS. However, the motor and pre-motor cortices are likely the nidus of origin and can be considered a local network node from which disease spreads out into the larger neocortical connectome. Identification of the site of origin of ALS is not merely of academic interest. There is a lengthy pre-clinical period in ALS [84], so the search for early markers of the disease is essential to rescue dysfunctional but not dead neurons. Markers need to take cognizance of cortical events in ALS. As precision medicine matures, directing attention to the site of origin of a disease becomes ever more relevant in terms of therapeutic endeavors.

## References

[1] Lemon R.N., Griffiths J. (2005). Comparing the function of the corticospinal system in different species: Organizational differences for motor specialization?. Muscle Nerve.

[2] Kiernan M.C., Vucic S., Cheah B.C., Turner M.R., Eisen A., Hardiman O., Burrell J.R., Zoing M.C. (2011). Amyotrophic lateral sclerosis. Lancet.

[3] Eisen A. (2009). Amyotrophic lateral sclerosis: A 40-year personal perspective. J. Clin. Neurosci..

[4] Lemon R.N. (2008). Descending pathways in motor control. Annu. Rev. Neurosci..

[5] Snowden J.S., Harris J., Richardson A., Rollinson S., Thompson J.C., Neary D., Mann D.M., Pickering-Brown S. (2013). Frontotemporal dementia with amyotrophic lateral sclerosis: A clinical comparison of patients with and without repeat expansions in C9orf72. Amyotroph. Lateral Scler. Front. Degener..

[6] Woolley S.C., Strong M.J. (2015). Frontotemporal Dysfunction and Dementia in Amyotrophic Lateral Sclerosis. Neurol. Clin..

[7] Eisen A., Kim S., Pant B. (1992). Amyotrophic lateral sclerosis (ALS): A phylogenetic disease of the corticomotoneuron?. Muscle Nerve.

[8] Eisen A., Braak H., Del Tredici K., Lemon R., Ludolph A.C., Kiernan M.C. (2017). Cortical influences drive amyotrophic lateral sclerosis. J. Neurol. Neurosurg. Psychiatry.

[9] Baker M.R. (2014). ALS—Dying forward, backward or outward?. Nat. Rev. Neurol..

[10] Braak H., Brettschneider J., Ludolph A.C., Lee V.M., Trojanowski J.Q., Del Tredici K. (2013). Amyotrophic lateral sclerosis—A model of corticofugal axonal spread. Nat. Rev. Neurol..

[11] Ludolph A.C., Emilian S., Dreyhaupt J., Rosenbohm A., Kraskov A., Lemon R.N., Del Tredici K., Braak H. (2020). Pattern of paresis in ALS is consistent with the physiology of the corticomotoneuronal projections to different muscle groups. J. Neurol. Neurosurg. Psychiatry.

[12] Eisen A., Weber M. (2001). The motor cortex and amyotrophic lateral sclerosis. Muscle Nerve.

[13] Braak H., Ludolph A.C., Neumann M., Ravits J., Del Tredici K. (2017). Pathological TDP-43 changes in Betz cells differ from those in bulbar and spinal alpha-motoneurons in sporadic amyotrophic lateral sclerosis. Acta Neuropathol..

[14] Vucic S., Kiernan M.C. (2006). Novel threshold tracking techniques suggest that cortical hyperexcitability is an early feature of motor neuron disease. Brain J. Neurol..

[15] Vucic S., Kiernan M.C. (2013). Utility of transcranial magnetic stimulation in delineating amyotrophic lateral sclerosis pathophysiology. Handb. Clin. Neurol..

[16] Vucic S., Ziemann U., Eisen A., Hallett M., Kiernan M.C. (2013). Transcranial magnetic stimulation and amyotrophic lateral sclerosis: Pathophysiological insights. J. Neurol. Neurosurg. Psychiatry.

[17] Turner M.R., Agosta F., Bede P., Govind V., Lule D., Verstraete E. (2012). Neuroimaging in amyotrophic lateral sclerosis. Biomark. Med..

[18] Brownell B., Oppenheimer D.R., Hughes J.T. (1970). The central nervous system in motor neurone disease. J. Neurol. Neurosurg. Psychiatry.

[19] Tu S., Wang C., Menke R.A.L., Talbot K., Barnett M., Kiernan M.C., Turner M.R. (2020). Regional callosal integrity and bilaterality of limb weakness in amyotrophic lateral sclerosis. Amyotroph. Lateral Scler. Front. Degener..

[20] Eisen A., Turner M.R., Lemon R. (2014). Tools and talk: An evolutionary perspective on the functional deficits associated with amyotrophic lateral sclerosis. Muscle Nerve.

[21] Charcot J. (1874). Sclerose laterale amytrophique. Oeuvres Compltes. Bur. Proges Med..

[22] Charcot J., Hadden W.B. (1883). Lectures on the Localization of Cerebral and Spinal Diseases.

[23] Kushchayev S.V., Moskalenko V.F., Wiener P.C., Tsymbaliuk V.I., Cherkasov V.G., Dzyavulska I.V., Kovalchuk O.I., Sonntag V.K., Spetzler R.F., Preul M.C. (2012). The discovery of the pyramidal neurons: Vladimir Betz and a new era of neuroscience. Brain.

[24] Betz W. (1874). Anatomischer Nachweis zweier Gehirncentra. Cent. Die Med. Wiss..

[25] Lewis B. (1878). On the comparitive structure of the cortex cerebri. Brain.

[26] Walshe F.M.R. (1942). The giant cells of Betz, the motor cortex and the prymaidal tract: A critical review. Brain.

[27] Walshe F.M.R. (1955). The pyamidal tract. Brain.

[28] Gowers W.R. (1893). A Manual of Diseases of the Nervous System.

[29] Mott F.W. (1895). A case of amyotrophc lateral sclerosis with degeneration of the motor path from cortex to the periphery. Brain J. Neurol..

[30] Wilson S.A. (1940). Kinnier: Neurology.

[31] Hudson A.J., Kiernan J.N. (1988). Preservation of certain voluntary muscles in motoneurone disease. Lancet.

[32] Kiernan J.A., Hudson A.J. (1991). Changes in sizes of cortical and lower motor neurons in amyotrophic lateral sclerosis. Brain J. Neurol..

[33] Rathelot J.A., Strick P.L. (2009). Subdivisions of primary motor cortex based on cortico-motoneuronal cells. Proc. Natl. Acad. Sci. USA.

[34] Witham C.L., Fisher K.M., Edgley S.A., Baker S.N. (2016). Corticospinal Inputs to Primate Motoneurons Innervating the Forelimb from Two Divisions of Primary Motor Cortex and Area 3a. J. Neurosci..

[35] Braak H., Braak E. (1976). The pyramidal cells of Betz within the cingulate and precentral gigantopyramidal field in the human brain. A Golgi and pigmentarchitectonic study. Cell Tissue Res..

[36] Kremer S., Chassagnon S., Hoffmann D., Benabid A.L., Kahane P. (2001). The cingulate hidden hand. J. Neurol. Neurosurg. Psychiatry.

[37] Sanides F. (1970). Evolutionary aspect of the primate neocortex. Proceedings of the International Primatological Society Third International Congress of Primatology.

[38] Sanides F., Sanides D. (1972). The “extraverted neurons” of the mammalian cerebral cortex. Z. Anat. Entwickl..

[39] Morecraft R.J., Ge J., Stilwell-Morecraft K.S., McNeal D.W., Pizzimenti M.A., Darling W.G. (2013). Terminal distribution of the corticospinal projection from the hand/arm region of the primary motor cortex to the cervical enlargement in rhesus monkey. J. Comp. Neurol..

[40] Ralston D.D., Ralston H.J. (1985). The terminations of corticospinal tract axons in the macaque monkey. J. Comp. Neurol..

[41] Graf von Keyserlingk D., Schramm U. (1984). Diameter of axons and thickness of myelin sheaths of the pyramidal tract fibres in the adult human medullary pyramid. Anat. Anz..

[42] Porter R., Lemon R.N. (1993). Corticospinal Neurones and Voluntary Movement.

[43] Iwatsubo T., Kuzuhara S., Kanemitsu A., Shimada H., Toyokura Y. (1990). Corticofugal projections to the motor nuclei of the brainstem and spinal cord in humans. Neurology.

[44] Fornia L., Ferpozzi V., Montagna M., Rossi M., Riva M., Pessina F., Martinelli Boneschi F., Borroni P., Lemon R.N., Bello L. (2016). Functional Characterization of the Left Ventrolateral Premotor Cortex in Humans: A Direct Electrophysiological Approach. Cereb. Cortex.

[45] Eisen A., Kuwabara S. (2012). The split hand syndrome in amyotrophic lateral sclerosis. J. Neurol. Neurosurg. Psychiatry.

[46] Menon P., Kiernan M.C., Vucic S. (2014). Cortical excitability differences in hand muscles follow a split-hand pattern in healthy controls. Muscle Nerve.

[47] Simon N.G., Lee M., Bae J.S., Mioshi E., Lin C.S., Pfluger C.M., Henderson R.D., Vucic S., Swash M., Burke D. (2015). Dissociated lower limb muscle involvement in amyotrophic lateral sclerosis. J. Neurol..

[48] Khalaf R., Martin S., Ellis C., Burman R., Sreedharan J., Shaw C., Leigh P.N., Turner M.R., Al-Chalabi A. (2019). Relative preservation of triceps over biceps strength in upper limb-onset ALS: The ‘split elbow’. J. Neurol. Neurosurg. Psychiatry.

[49] Vucic S. (2019). Split elbow sign: More evidence for the importance of cortical dysfunction in ALS. J. Neurol. Neurosurg. Psychiatry.

[50] Lemon R.N., Landau W., Tutssel D., Lawrence D.G. (2012). Lawrence and Kuypers (1968a, b) revisited: Copies of the original filmed material from their classic papers in Brain. Brain J. Neurol..

[51] Kuypers H., Brookhart J., Mountcastle V.B. (1981). Anatomy of the descending pathways. Handbook of Physiology—The Nervous System II.

[52] Marinacci A.A., VonHagen K.O. (1974). Electromyography in amyotrophic lateral sclerosis. A review. Bull. Los Angel. Neurol. Soc..

[53] Daube J.R. (1985). Electrophysiologic studies in the diagnosis and prognosis of motor neuron diseases. Neurol. Clin..

[54] Swash M. (2012). Why are upper motor neuron signs difficult to elicit in amyotrophic lateral sclerosis?. J. Neurol. Neurosurg. Psychiatry.

[55] Swash M., Burke D., Turner M.R., Grosskreutz J., Leigh P.N., deCarvalho M., Kiernan M.C. (2020). Occasional essay: Upper motor neuron syndrome in amyotrophic lateral sclerosis. J. Neurol. Neurosurg. Psychiatry.

[56] van Es M.A., Goedee H.S., Westeneng H.J., Nijboer T.C.W., van den Berg L.H. (2020). Is it accurate to classify ALS as a neuromuscular disorder?. Expert Rev. Neurother..

[57] Eisen A., Calne D. (1992). Amyotrophic lateral sclerosis, Parkinson’s disease and Alzheimer’s disease: Phylogenetic disorders of the human neocortex sharing many characteristics. Can. J. Neurol. Sci..

[58] Kang B.H., Kim J.I., Lim Y.M., Kim K.K. (2018). Abnormal Oculomotor Functions in Amyotrophic Lateral Sclerosis. J. Clin. Neurol..

[59] Carvalho M., Schwartz M.S., Swash M. (1995). Involvement of the external anal sphincter in amyotrophic lateral sclerosis. Muscle Nerve.

[60] Goodin D.S., Rowley H.A., Olney R.K. (1988). Magnetic resonance imaging in amyotrophic lateral sclerosis. Ann. Neurol..

[61] Iwasaki Y., Kinoshita M., Ikeda K., Takamiya K. (1989). Central nervous system magnetic resonance imaging findings in amyotrophic lateral sclerosis. Eur. Arch. Psychiatry Neurol. Sci..

[62] Turner M.R., Verstraete E. (2015). What does imaging reveal about the pathology of amyotrophic lateral sclerosis?. Curr. Neurol. Neurosci. Rep..

[63] Mills K.R., Murray N.M., Hess C.W. (1987). Magnetic and electrical transcranial brain stimulation: Physiological mechanisms and clinical applications. Neurosurgery.

[64] Eisen A.A., Shtybel W. (1990). AAEM minimonograph #35: Clinical experience with transcranial magnetic stimulation. Muscle Nerve.

[65] Eisen A., Shytbel W., Murphy K., Hoirch M. (1990). Cortical magnetic stimulation in amyotrophic lateral sclerosis. Muscle Nerve.

[66] Brown W.F., Ebers G.C., Hudson A.J., Pringle C.E., Veitch J. (1992). Motor-evoked responses in primary lateral sclerosis. Muscle Nerve.

[67] Vucic S., Howells J., Trevillion L., Kiernan M.C. (2006). Assessment of cortical excitability using threshold tracking techniques. Muscle Nerve.

[68] Vucic S., Nicholson G.A., Kiernan M.C. (2008). Cortical hyperexcitability may precede the onset of familial amyotrophic lateral sclerosis. Brain J. Neurol..

[69] van der Graaff M.M., de Jong J.M., Baas F., de Visser M. (2009). Upper motor neuron and extra-motor neuron involvement in amyotrophic lateral sclerosis: A clinical and brain imaging review. Neuromuscul. Disord..

[70] Agosta F., Chio A., Cosottini M., De Stefano N., Falini A., Mascalchi M., Rocca M.A., Silani V., Tedeschi G., Filippi M. (2010). The present and the future of neuroimaging in amyotrophic lateral sclerosis. Ajnr. Am. J. Neuroradiol..

[71] Mezzapesa D.M., D’Errico E., Tortelli R., Distaso E., Cortese R., Tursi M., Federico F., Zoccolella S., Logroscino G., Dicuonzo F. (2013). Cortical thinning and clinical heterogeneity in amyotrophic lateral sclerosis. PLoS ONE.

[72] Turner M.R., Modo M. (2010). Advances in the application of MRI to amyotrophic lateral sclerosis. Expert Opin. Med. Diagn..

[73] Lillo P., Hodges J.R. (2009). Frontotemporal dementia and motor neurone disease: Overlapping clinic-pathological disorders. J. Clin. Neurosci..

[74] Neary D., Snowden J. (2013). Frontal lobe dementia, motor neuron disease, and clinical and neuropathological criteria. J. Neurol. Neurosurg. Psychiatry.

[75] Hudson A.J. (1981). Amyotrophic lateral sclerosis and its association with dementia, parkinsonism and other neurological disorders: A review. Brain J. Neurol..

[76] Hudson A.J., Martzke J. (1993). Aphasic dementia and motor neuron disease. Ann. Neurol..

[77] Strong M.J. (2008). The syndromes of frontotemporal dysfunction in amyotrophic lateral sclerosis. Amyotroph. Lateral Scler..

[78] DeJesus-Hernandez M., Mackenzie I.R., Boeve B.F., Boxer A.L., Baker M., Rutherford N.J., Nicholson A.M., Finch N.A., Flynn H., Adamson J. (2011). Expanded GGGGCC hexanucleotide repeat in noncoding region of C9ORF72 causes chromosome 9p-linked FTD and ALS. Neuron.

[79] Renton A.E., Majounie E., Waite A., Simon-Sanchez J., Rollinson S., Gibbs J.R., Schymick J.C., Laaksovirta H., van Swieten J.C., Myllykangas L. (2011). A hexanucleotide repeat expansion in C9ORF72 is the cause of chromosome 9p21-linked ALS-FTD. Neuron.

[80] Uddin L.Q., Yeo B.T.T., Spreng R.N. (2019). Towards a Universal Taxonomy of Macro-scale Functional Human Brain Networks. Brain Topogr..

[81] Verstraete E., van den Heuvel M.P., Veldink J.H., Blanken N., Mandl R.C., Hulshoff Pol H.E., van den Berg L.H. (2010). Motor network degeneration in amyotrophic lateral sclerosis: A structural and functional connectivity study. PLoS ONE.

[82] Buchanan C.R., Pettit L.D., Storkey A.J., Abrahams S., Bastin M.E. (2015). Reduced structural connectivity within a prefrontal-motor-subcortical network in amyotrophic lateral sclerosis. J. Magn. Reson. Imaging.

[83] Ravits J.M., La Spada A.R. (2009). ALS motor phenotype heterogeneity, focality, and spread: Deconstructing motor neuron degeneration. Neurology.

[84] Eisen A., Kiernan M., Mitsumoto H., Swash M. (2014). Amyotrophic lateral sclerosis: A long preclinical period?. Neurol. Neurosurg. Psychiatry.

